# HIV Co-receptor usage in HIV-related non-hodgkin's lymphoma

**DOI:** 10.1186/1750-9378-7-6

**Published:** 2012-03-15

**Authors:** Erin Reid, Sheldon R Morris

**Affiliations:** 1 Department of Medicine, Division of Hematology and Oncology, University of California, San Diego, Room 2238, 3855 Health Sciences Drive, La Jolla, San Diego, CA 92093-0987, USA; 2 Department of Medicine, Division of Infectious Diseases, University of California, San Diego, 150 West Washington St, San Diego, CA 92103, USA

**Keywords:** HIV, Non-hodgkins lymphoma, Co-receptor usage, CCR5 CXCR4, SDF-1

## Abstract

In this study 15 banked samples of HIV-related Non-Hodgkin's Lymphoma (NHL) cases were tested for HIV co-receptor usage and SDF1 3'A polymorphism. Reportable tropism from 9 plasma samples had 1 (11.1%) HIV case with CXCR4 and 8 (88.9%) with CCR5 usage, even though most of the cases occurred at a late stage of HIV (2/3 had CD4 counts below 200), where expected CXCR4 usage would be 60%. Based on the expected proportion of less than 50% CCR5 in chronically infected individuals, this would suggest that in NHL may be associated with CCR5 usage (*P *= 0.04).

## Introduction

Highly active antiretroviral treatment (HAART) has prolonged survival in HIV infected individuals [1], however, HIV associated malignancies remain relatively common [2]. Lymphoma is a frequent HIV-related malignancy [3], that generally presents in late HIV disease, possibly related to worsening immune function that allows latent Epstein Barr Virus (EBV) in memory B cells to generate a proliferative condition [4,5]. We considered what HIV factors may also play a role in lymphoma and noted that with later HIV disease stages the virus acquires CXCR4 co-receptor usage [6]. In early stage HIV infection, at most, only about 15% are dual/mixed (DM) tropic for CCR5 and CXCR4 [7,8], then in late disease stages, DM tropism or pure CXCR4 tropism reaches up to 60% when measured by the enhanced tropism assay [9], and is the highest with CD4 ≤ 200 cells/ml [10,11]. The chemokine receptor CXCR4 is also highly expressed in hematological malignancies [12,13]. The chemokine for CXCR4, stromal cell derived factor 1 (SDF1), has also been associated with HIV associated Non-Hodgkin's lymphoma (NHL) when individuals have a polymorphism in the SDF1 gene from G to A transition at position 801 (SDF1-3'A) that increased with homozygosity [14]. In North America, SDF1-3'A is expected in 21% of Caucasians [15]. Although there was some suggestion that SDF1-3'A is associated with HIV disease progression [16], and CXCR4 tropic virus [17]. Based on these observations, we examined whether HIV co-receptor usage and the SDF1 polymorphisms were associated with HIV-related NHL.

## Methods

A study protocol was submitted and approved to the AIDS and Cancer Specimen Resource (ACSR) to supply 16 samples of NHL from HIV-infected individuals that had plasma and PBMC samples at a time of viral load greater than 1000, the threshold required for the Trofile assay (Monogram Biosciences Inc.). An IRB approval was obtained to collect and test these samples with diagnosis, demographics, HIV viral load, CD4 count for each subject. Samples were shipped from two ACSR repositories on dry ice and were processed for plasma to be sent for Trofile ES assay. The PBMCs were processed at the UCSD Center for AIDS Research Genomics Core laboratory. SDF1 polymorphisms were done with SNP analysis for the SDF 1-3'A polymorphism (Applied Biosystems). Data analysis employed SAS v 9.2 statistical software to describe the frequencies of HIV co-receptor tropism, SDF1-3'A, demographics, CD4 counts and HIV viral loads at time of the samples. Rates of expected proportions were compared with in a one-sample binomial proportion test for statistical significance in SAS v9.2.

## Results

There were 15 paired samples from HIV-infected individuals with confirmed NHL according to the ACSR database. Cases of HIV-related NHL were mainly male (93%) with median age of 47 (Table 1) and CD4 count ≤ 200 cells/ml in 9 (60%) cases. The median CD4 count was 148 (range 5-1056) and the median log_10 _viral load was 4.54 log copies/ml (range 3.32-5.88). Trofile assays were performed on all samples but 6 of the samples failed to culture and were non-reportable. Only one reportable tropism had DM tropism (11.1%, 95% Exact confidence interval 0.3-48.2%) and eight were CCR5 (88.9%, 95% Exact confidence interval (CI) 68.3-100%). Based on an expected estimate of 50% CCR5 use in late stage HIV-infected individuals the proportion of CCR5 tropism in the NHL samples was higher that expected ((*P *= 0.04). Tropism reportable cases had CD4 counts with median of 148 cells/ml (range 5-821). Those with reportable tropism did not differ in CD4 and viral load to those without a reportable tropism (Wilcoxon two sample t approximation *p = *0.39 and *p = *0.95). All PBMC samples were testing for the SDF polymorphism and four were heterozygous for the SDF1-3'A allele (26.7%, 95% CI 7.8-55.1). This rate of SDF1-3'A was no greater than expected in the general population based on one sided binomial proportion test against a base proportion of 21% (*P *= 0.39). Of the samples that had reportable tropism there were three (33.3%) with SDF-3'A heterozygosity. The one DM tropism was a SDF-3'A heterozygote compared to 25% of CCR5 tropism.

**Table 1:** HIV Co-receptor Tropism and SDF1 3'A among Individuals with Non-Hodgkin's Lymphoma

	All	Tropism Reportable
N	15	9

Male (%)	14 (93.3)	8 (88.9)

Age median (range)	47 (36-60)	47 (37-60)

Median CD4 Count (range)	148 (5-1056)	148 (5-821)

Median log10 Viral Load (range)	4.54 (3.32-5.89)	4.00 (3.63-5.39)

HIV co-receptor tropism (%)		
CCR5	8 (53.3)	8 (88.9)
Dual Mixed (CCR5 and CXCR4)	1 (6.7)	1 (11.1)
CXCR4	0	0 (0)
Not reportable	6 (40.0)	--

SDF1 polymorphism^1 ^(%)		
WT/WT	11 (73.3)	6 (66.7)
WT/3'A	4 (26.7)	3 (33.3)
3'A/3'A	0 (0)	0 (0)

^1^WT = wild type

## Discussion

This is the first data that described the viral co-receptor tropism in HIV-infected subjects with NHL. The finding of only one of nine subjects had CXCR4 co-receptor usage using the most sensitive assay was lower than would have been expected and suggests a preponderance of CCR5 use in the NHL subjects. In our cases, SDF1-3'A was not significantly higher than the expected population levels. Although SD1-3'A was found with the only CXCR4 tropism, there was not enough outcomes to verify an association. Contrary to our initial hypothesis, our finding suggests, if there exists a relationship of NHL with HIV co-receptor, it will be with persistent CCR5 usage, but further studies are needed to validate this association.

## Competing interests

The authors declare that they have no competing interests.

## Authors' contributions

Both authors (ER and SM) contributed substantively to the design, acquisition of data, and drafting of the manuscript. ER arranged for the samples to be available. SM oversaw the sample processing and testing. SM did the analysis and was the primary authorship of the paper. All authors read and approved the final manuscript.
